# Comparison of Thalamus and Basal Ganglia Signs Between Multiple Sclerosis and Primary Angiitis of the Central Nervous System: An Exploratory Study

**DOI:** 10.3389/fneur.2021.513253

**Published:** 2021-07-29

**Authors:** Ying Chen, Rui Li, Aimin Wu, Wei Qiu, Xueqiang Hu, Zhaoqi Hu, Qian Yang, Zhiming Zhou

**Affiliations:** ^1^Department of Neurology, Yijishan Hospital, The First Affiliated Hospital of Wannan Medical College, Wuhu, China; ^2^Department of Neurology, The Third Affiliated Hospital of Sun Yat-Sen University, Guangzhou, China; ^3^Department of Orthopaedics, Wuhu Traditional Chinese Medicine Hospital, Wuhu, China

**Keywords:** multiple sclerosis, primary angiitis of the central nervous system, basal ganglia, MRI, comparison

## Abstract

Based on the symptoms, especially those affecting small vessels, it is difficult to distinguish multiple sclerosis (MS) from primary angiitis of the central nervous system (PACNS). Magnetic resonance imaging (MRI) helps understand the characteristics of deep gray matter lesions (DGML) in MS and PACNS. We aimed to compare the MRI characteristics of thalamus and basal ganglia lesions between relapsing-remitting MS and PACNS. In our study, 49 relapsing-remitting MS patients and 16 PACNS with MRI-confirmed thalamus or basal ganglia lesions were enrolled. Among the DGMLs in basal ganglia, putamen had significantly higher (*P* = 0.037) involvement in PACNS than in MS. More importantly, larger lesion sizes in thalamus helps to distinguish PACNS (12.4 ± 4.3 mm) from MS (7.9 ± 3.7 mm) (*P* = 0.006). But using lesions in basal ganglia, researchers were unable to differentiate the two disorders. Presently, our study shows that MRI performances of deep gray matter differ between MS and PACNS.

## Introduction

Multiple sclerosis (MS) is a chronic inflammatory demyelinating disease of the central nervous system (CNS), which is characterized by recurrent neurological deficits, attributed to lesions scattered in the brain and spinal cord (1). The involvement of deep gray matter (GM) structures in MS is of particular interest, because the thalamus, limbic, and striatal structures are involved in all the major functional circuits in the brain and provide points of convergence across multiple cortical, limbic, brain stem, and cerebellar systems (2). Lesions in these regions are important components of MS pathology. Gray matter demyelination has been documented in the hippocampus, basal ganglia, and thalamus, and can be observed by magnetic resonance imaging (MRI) (3, 4). Deep gray matter abnormality measures on MRI scans have been correlated with disability and cognitive impairment (5, 6).

Primary angiitis of the central nervous system (PACNS) is a rare idiopathic disorder, which causes inflammation in small and medium-sized vessels (7). Patients with PACNS sometimes exhibit “MS-like” relapsing-remitting or progressive symptoms when they are affected in small vessels in acute stage (8, 9). Deep gray matter can also be impaired in PACNS. A study in 2016 indicated 74% of PACNS sufferers exhibited lesions in deep gray matter (10). Although these lesions could be visualized by MRI, they tend to provide the topographic MRI evidence to make a diagnosis of MS, leading to misdiagnosis (9). In this situation, the lack of a clear differentiation between MS and PACNS can be problematic.

**Figure 1:**
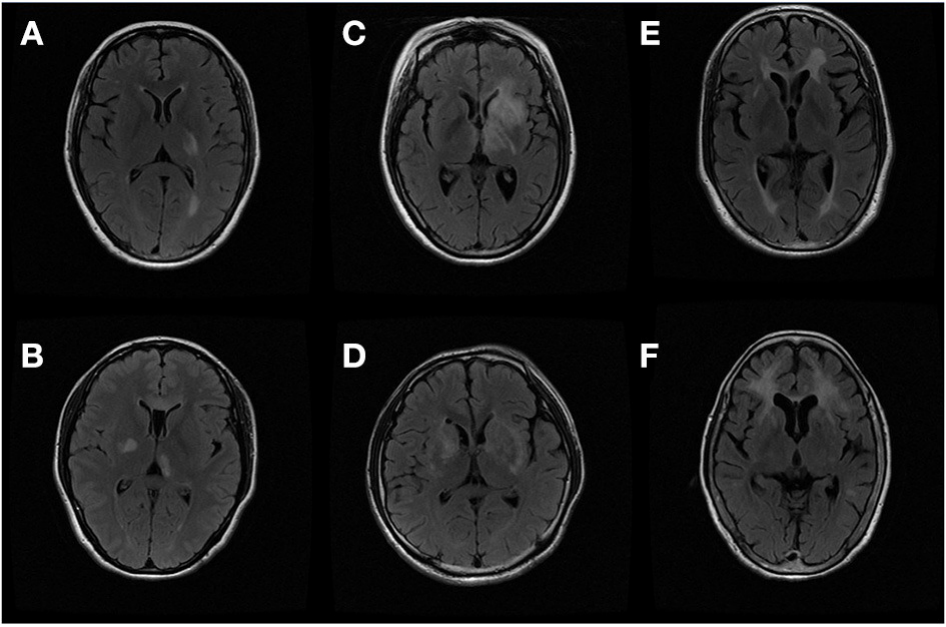
Thalamus and basal ganglia lesions on magnetic resonance image (MRI): **(A,B)** thalamus lesions of multiple sclerosis (MS) and primary central nervous system angiitis (PACNS); **(C,D)** internal capsule lesions of MS and PACNS; **(E,F)** putamen lesions of MS and PACNS.

We then made a deep investigation of MRI characteristics of basal ganglia and thalamus lesions in MS and PACNS, and compared them in this study (Figure 1).

## Methods

### Ethics Statement

This study was approved by the local Ethics Committee of the Third Affiliated Hospital of Sun Yat-sen University (NO 2011-2-48). Informed consent for this investigation was obtained from the patients to evaluate the examination scores and standard laboratory test results that benefited the diagnosis and therapy.

### Patients

We retrospectively reviewed 49 patients diagnosed with MS and 16 patients with PACNS from November 2013 to October 2018 in the MS center of the Third Affiliated Hospital of Sun Yat-sen University and the First Affiliated Hospital of Wannan Medical University. All of the patients included were adults. PACNS was diagnosed in accordance with Calabrese and Mallek in 1988 (11), and required: the presence of an acquired otherwise unexplained neurological or psychiatric deficit, the presence of either classic angiographic or histopathological features of angiitis within the CNS, and no evidence of systemic vasculitis or any disorder that could cause or mimic the angiographic or pathological features of the disease. Among them, only two patients underwent brain biopsy while the rest chose less invasive angiography. They did not meet the 2010 McDonald criteria for MS (12). The patients diagnosed with MS were from the subgroup of relapsing-remitting MS according to the 2010 McDonald criteria. Our research enrolled 49 relapsing-remitting MS patients and 16 PACNS with MRI-confirmed thalamus or basal ganglia lesions. All of the patients were followed up for at least 12 months. None of the patients had systemic or inflammatory diseases elsewhere besides the central nervous system. All of the patients were given such diagnoses initially and had not received any corticosteroid before their enrollment. The decision of whether to take immunomodulatory drugs was carefully made after a thorough examination of the patients.

### MRI Acquisition Protocol

A 1.5-T magnetic resonance imager (General Electric, Milwaukee, WI, USA) was used to perform MRI of the brain and spinal cord. Conventional MRI protocols were applied to all the patients: T1-weighted images (T1W) with and without gadolinium enhancement (GDE), T2-weighted images (T2W), and fluid attenuated inversion recovery (FLAIR). The diameters of lesions with T2 contrast enhancement, black holes, and confluency were measured on axial sections with T2-FLAIR sequences. Patients were excluded if contraindicated for MRI or intravenous injection of gadolinium-based contrast material. MR angiography was also applied to all patients. All image archives were reviewed with a DICOM viewer on a Macintosh computer. An experienced neurologist and neuroradiologist, who were both blinded to the grouping of the two diseases and patients' clinical features, analyzed all MRI images. The final results were resolved by discussion.

### Statistical Analysis

Quantitative data in this study were analyzed with independent *t*-test for normally distributed variables and Mann–Whitney *U*-test for non-normally distributed variables. Results were presented as mean ± standard deviation (SD) or median ± range. Our quantitative data is presented with $\bar{x}$ ± s. The independent sample *t*-test or corrected *t*-test (when the variance was uneven) was used for comparison between groups. The data that did not conform to the normal distribution was tested by the Mann-Whitney *U*-test, and the qualitative data was tested by the chi-square test or Fisher's exact test. No multiple comparison was applied, and the test standard was *P* < 0.05 for statistical difference. Statistical analysis was performed by using SPSS 21.0 (SPSS Inc, Chicago, IL, USA).

## Results

### Clinical Characteristics

Demographic and clinical features of 65 patients in our study were displayed in Table 1. There was no significant difference in onset age and gender distribution between MS and PACNS (*P* > 0.05). Patients with PACNS (43.8%) presented with headache more often than MS (11.4%) (*P* = 0.003). Visual impairment was found in 21 MS patients (42.9%), but only in two PACNS sufferers (12.5%) (*P* = 0.036). Sensory disorder (*P* = 0.761) and limb weakness (*P* = 0.146) were more prevalent in MS, but without differences, compared with PACNS.

**Table 1:** Clinical features of multiple sclerosis (MS) and primary angiitis of central nervous system (PACNS).

	**MS (*n* = 49)**	**PACNS (*n* = 16)**	***p***
Female percentage (%)	24 (48.5%)	8 (50%)	0.943
Age at onset, years (SD)	35.0 ± 13.1	32.0 ± 18.3	0.476
Range (years)	19-63	20-70	
Disease duration (months)	33.9 ± 19.13	13.7 ± 9.39	0.003
Range	6-84	1-24	
Follow-up (months)	25.3 ± 9.8	22.5 ± 9.0	0.501
Range	12-43	14-40	
Relapses (SD)	3 ± 1.5	2 ± 1.2	_
**Clinical features**			
Headache (%)	5 (11.4%)	7 (43.8%)	0.003
Visual impairment (%)	21 (42.9%)	2 (12.5%)	0.036
Sensory symptoms (%)	15 (30.6%)	4 (25.0%)	0.761
Limb weakness (%)	24 (48.5%)	4 (25%)	0.146

### MRI Features

As presented in Table 2, lesions showed more occurrence in PACNS cases, rather than in MS patients, in the regions of the putamen (68.8 vs. 38.8%) with significant difference (*P* = 0.037). For other areas, no differences were found between the two groups (*P* > 0.05).

**Table 2:** Comparison of basal ganglia/thalamus lesions between multiple sclerosis (MS) and primary angiitis of central nervous system (PACNS).

	**MS**	**PACNS**	***p***
**Cases involved**, ***n*****(%)**			
Putamen	19 (38.8%)	11 (68.8%)	0.037
Caudatum	8 (16.3%)	2 (12.5%)	1.000
Globus pallidus	9 (18.4%)	5 (31.3%)	0.276
Claustrum	3 (6.1%)	2 (12.5%)	0.590
Thalamus	41 (83.7%)	7 (43.8%)	0.002
Internal capsule	27 (55.1%)	6 (37.5%)	0.221
External capsule	8 (16.3%)	4 (25.0%)	0.470
**Diameter, mm (SD)**			
Putamen	9.3 ± 5.4	11.3 ± 8.5	0.437
Caudatum	9.6 ± 4.3	6.0 ± 2.8	0.299
Globus pallidus	6.4 ± 3.9	6.4 ± 5.0	0.986
Thalamus	7.9 ± 3.7	12.4 ± 4.3	0.006
Internal capsule	6.4 ± 3.9	11.9 ± 6.1	0.055
External capsule	13.6 ± 6.0	6.8 ± 1.5	0.053

We then examined the overall size of lesions and found that size of lesion in MS was much smaller than that in PACNS (*P* = 0.004). Considering the regions, MS had obviously smaller thalamus lesions than PACNS (MS vs. PACNS 7.9 ± 3.7 mm vs. 12.4 ± 4.3 mm, *P* = 0.006). The diameter of the lesions located in putamen of MS was smaller than those from PACNS, but without any significance (MS vs. PACNS 9.3 ± 5.4 mm vs. 11.3 ± 8.5 mm, *P* = 0.437). There was also no significance between the two diseases, with respect to caudatum (MS vs. PACNS 9.6 ± 4.3 mm vs. 6.0 ± 2.8 mm, *P* = 0.299) and globus pallidus (MS vs. PACNS 6.4 ± 3.9 mm vs. 6.4 ± 5.0 mm, *P* = 0.986). MS patients tended to have smaller lesions than PACNS patients in internal capsule (MS vs. PACNS 6.4 ± 3.9 mm vs. 11.9 ± 6.1 mm, *P* = 0.055), but with a low significance.

## Discussion

This study evaluated MRI characteristics of deep gray matter lesions in relapsing-remitting MS and PACNS. The results provided new radiological evidence for distinguishing MS from PACNS.

Although MS and PACNS are two different diseases considering their causes and etiologies, they can both express a chronic relapsing-remitting or progressive clinical course with MS-like symptoms, as well as brain abnormalities that are hard to distinguish from each other (9, 13). Moreover, PACNS remains challenging to diagnose, especially when small vessels are involved. So the value in this study is in helping to analyze the MRI characteristics in deep gray matter.

Deep gray matter is an important structure in the brain and is often affected in both Diseases (10, 14–16). Recently, the involvement of gray matter regions in MS demyelinating process has aroused much attention. In our study, the majority of DGML in basal ganglia had higher involvement in vasculitis than MS. Among them, the putamen was significantly more involved in primary CNS vasculitis. The insignificant variance in distribution of internal capsule lesions between the two groups might be a little perplexing at first. However, high involvement of internal capsule in MS and its correlation with disability, proven by Dalton et al. (17), made our results reasonable. Compared with PACNS patients, there is a higher number of thalamic lesions among patients with MS. However, this result should be interpreted with caution. Because we specifically selected MS patients with thalamic and/or basal ganglia lesions, not consecutive patients, the frequency of lesions' (e.g., thalamic lesions) appearance may not reflect the actual frequency of lesions' appearance. A further well-designed study with consecutive recruitment of patients is needed.

Thalamus and basal ganglia are also targeted by MS according to a number of studies (18, 19). Significant lymphocyte infiltration, complement deposition, and blood–brain barrier (BBB) disruption have so far not been detected in gray matter lesions, which makes it distinctive from the inflammatory process in white matter lesions (20). The less severe or even exemption of inflammation and BBB disruption in these regions makes the foci diameter in this location limited. This in a way offers an explanation for smaller lesions in the thalamus when compared with PACNS. Though the putamen was more frequently involved in vasculitis, the average diameter in MS was not distinctively smaller than in PACNS. The almost equal size of lesions in the internal and external gives us a hint that lesions from the basal ganglia might not be useful in making a differential diagnosis of MS and PACNS.

Also, there are several limitations to this study: (1) The number of PACNS patients in our study is small for the low morbidity of PACNS (2.4/million). Therefore, statistical deviation might happen; (2) There is inevitable bias caused by retrospective research; (3) No correction for multiple comparisons was performed in our study, so there is an increased risk for type 1 error; and (4) Selecting patients with thalamic and/or basal ganglia lesions does not allow for comparing the frequency of such lesions. This could be addressed in future studies by performing a consecutive recruitment of patients.

In conclusion, our study showed that involvement of the thalamus and putamen was useful in making differential diagnoses of MS and PACNS. MS tended to be affected in the thalamus, with smaller lesion sizes than PACNS. Since our study is an exploratory study, further studies are warranted to investigate the intrinsic mechanisms.

## Data Availability Statement

All datasets generated for this study are included in the article/supplementary material.

## Ethics Statement

This study was approved by the local Ethics Committee of the Third Affiliated Hospital of Sun Yat-sen University (NO 2011-2-48). Informed consent for this investigation was obtained from the patients to evaluate the examination scores and standard laboratory test results that benefited the diagnosis and therapy.

## Author Contributions

YC and AW designed the study. YC, RL, and ZH performed the study. YC, WQ, and XH wrote the article. RL, AW, QY, and ZZ helped revise the manuscript. All authors contributed to the article and approved the submitted version.

## Conflict of Interest

The authors declare that the research was conducted in the absence of any commercial or financial relationships that could be construed as a potential conflict of interest.

## Publisher's Note

All claims expressed in this article are solely those of the authors and do not necessarily represent those of their affiliated organizations, or those of the publisher, the editors and the reviewers. Any product that may be evaluated in this article, or claim that may be made by its manufacturer, is not guaranteed or endorsed by the publisher.
